# The Role of Epidermal Growth Factor Receptor Signaling Pathway during Bovine Herpesvirus 1 Productive Infection in Cell Culture

**DOI:** 10.3390/v12090927

**Published:** 2020-08-24

**Authors:** Wencai Qiu, Long Chang, Yongming He, Liqian Zhu

**Affiliations:** 1 College of Life Science and Engineering, Foshan University, Foshan 528231, China; wcqiu0909@163.com; 2 College of Veterinary Medicine, Yangzhou University, Yangzhou 225009, China; 18252789754@163.com; 3 Jiangsu Co-innovation Center for Prevention and Control of Important Animal Infectious Diseases and Zoonoses, Yangzhou 225009, China

**Keywords:** bovine herpesvirus 1, epidermal growth factor receptor, Akt, phospholipase C-γ1

## Abstract

Accumulating studies have shown that the epidermal growth factor receptor (EGFR) signaling pathway plays an essential role in mediating cellular entry of numerous viruses. In this study, we report that bovine herpesvirus 1 (BoHV-1) productive infection in both the human lung carcinoma cell line A549 and bovine kidney (MDBK) cells leads to activation of EGFR, as demonstrated by the increased phosphorylation of EGFR at Tyr1068 (Y1068), which in turn plays important roles in virus infection. A time-of-addition assay supported that virus replication at post-entry stages was affected by the EGFR specific inhibitor Gefitinib. Interestingly, both phospholipase C-γ1 (PLC-γ1) and Akt, canonical downstream effectors of EGFR, were activated following virus infection in A549 cells, while Gefitinib could inhibit the activation of PLC-γ1 but not Akt. In addition, virus titers in A549 cells was inhibited by chemical inhibition of PLC-γ1, but not by the inhibition of Akt. However, the Akt specific inhibitor Ly294002 could significantly reduce the virus titer in MDBK cells. Taken together, our data suggest that PLC-γ1 is stimulated in part through EGFR for efficient replication in A549 cells, whereas Akt can be stimulated by virus infection independent of EGFR, and is not essential for virus productive infection, indicating that Akt modulates BoHV-1 replication in a cell type-dependent manner. This study provides novel insights on how BoHV-1 infection activates EGFR signaling transduction to facilitate virus replication.

## 1. Introduction

Bovine herpesvirus 1 (BoHV-1) is a member of the family *Herpesviridae* and the subfamily *Alphaherpesvirinae*. BoHV-1 acute infection causes inflammatory disease in the upper respiratory tract, lesions on mucosal surfaces, and depression of host immune responses, which can facilitate secondary infection by other pathogens [1,2,3]. Consequently, this can result in bovine respiratory disease complex (BRDC), a life-threatening disease that affects cattle of all ages [4]. Early statistical data indicates that BRDC costs the U.S. cattle industry ~$3 billion annually [5]. Additionally, BoHV-1 is the most frequently diagnosed pathogen associated with viral abortion in the North American cattle industry [4,6]. BoHV-1 may cause abortion storms in a herd, with 25–60% of cows undergoing abortion [4]. As such, BoHV-1 infection represents an important disease in cattle.

Humans are not susceptible to BoHV-1 infection. However, since 2010, the virus has been shown to productively infect human cancer cells, such as the human lung adenocarcinoma cell line A549, inducing cell death [7,8,9]. This important finding suggests that BoHV-1 may represent a prospective reagent for cancer therapy [7]. Moreover, it provides us an alternative cell model for the comparative study of viral replication mechanisms in diverse cell cultures.

Epidermal growth factor receptor (EGFR) is a glycoprotein with a molecular weight of ~170 kDa that consists of an extracellular receptor domain, a transmembrane domain, and an intracellular domain with protein–tyrosine kinase activity [10]. Apart from the full-length EGFR protein, isoforms with distinct molecular weights, including 110, 80, and 60 kDa resulting either from alternative splicing or proteolytic cleavage of the receptor are known to exist [11]. Binding of EGFR to its cognate ligands leads to phosphorylation of the protein–tyrosine kinase domain and subsequent activation of downstream effectors, such as PLC-γ1 and Akt, to perform diverse functions [12,13]. Increasing evidence indicates that EGFR is activated by various viruses, leading to multiple pathogenic effects [14]. In example, EGFR kinase is activated by influenza A virus and hepatitis B virus to promote virus entry [15,16], and EGFR signaling is usurped during respiratory syncytial virus (RSV) infection to suppress IFN regulatory factor (IRF) 1-dependent CXCL10 production, resulting in suppression of host antiviral signaling [17]. Additionally, both EGFR/Erk1/2 and EGFR/STATs cascades are mobilized to control epithelial inflammatory responses following rhinovirus infection [18]. However, the effects of EGFR on BoHV-1 infection are currently unknown. Given that the canonical EGFR downstream effects including PLC-γ1, MAPK, Akt and JNK are unanimously activated during BoHV-1 productive infection in MDBK cells [19,20], we hypothesized that BoHV-1 infection activates EGFR signaling to facilitate viral replication. Furthermore, it has been reported that EGFR is activated in response to radiation-induced DNA damage to promote cell survival [21], and in BoHV-1-infected A549 cells, detectable DNA damage is induced at 24 and 48 hours (h) after infection [22]. As such, this work supports that EGFR is concomitantly activated at late stages of infection. Considering that EGFR signaling plays a key role in lung cancer progression, and that EGFR-targeted therapies represent effective approaches for the clinical treatment of lung tumors [23,24,25,26], studying the interplay between BoHV-1 infection and EGFR signaling with an emphasis on A549 cells is important not only in understating virus replication mechanisms but also in elucidating its oncolytic mechanisms.

Here, for the first time we report that EGFR signaling is activated in BoHV-1-infected cell cultures (A549 and MDBK cells), which in turn play important roles in subsequent virus replication. For efficient replication, PLC-γ1 is stimulated in BoHV-1-infected A549 cells partially through EGFR. However, Akt, a canonical EGFR downstream signaling mediator, is activated in A549 cells with an EGFR-independent mechanism, contributing only a limited role in virus replication. These findings add to our knowledge of how BoHV-1 manipulates EGFR and its downstream pathways for efficient replication in different cell cultures.

## 2. Materials and Methods

### 2.1. Cells and Viruses

A549 cells (purchased from the Chinese model culture preservation center, Shanghai, China) were maintained in DMEM medium (Thermo Fisher Scientific Inc, Waltham, MA, USA) supplemented with 10% fetal bovine serum (FBS) (Thermo Fisher Scientific Inc., Waltham, MA, USA, cat # 10270-106). MDBK cells (purchased from the Chinese model culture preservation center, Shanghai, China) were maintained in DMEM supplemented with 10% FBS. BoHV-1 (NJ-16-1 isolated from bovine semen samples [27]) was propagated in MDBK cells. Aliquots of virus stocks were stored at −70 °C until use.

### 2.2. Antibodies and Reagents

Phospho-EGFR-Y1068 monoclonal Ab (mAb) (cat# 2234S), Akt mAb (cat# 9272S), phospho-Akt (Ser473) mAb (cat# 9271S), phospho-PLC-γ1(Ser1248) mAb (cat# 8713), PLC-γ1 pAb (cat# 2822S), glyceraldehyde-3-phosphate dehydrogenase (GAPDH) mAb (cat# 2118), HRP labeled goat anti-rabbit IgG and goat anti-mouse IgG were purchased from Cell Signaling Technology (Danvers, MA, USA). EGFR polyclonal antibody (pAb) (cat# A11577) and β-Tubulin Rabbit pAb (cat# AC015) were provided by ABclonal Technology (Woburn, MA, USA). All the primary antibodies employed for Western blots were at a 1:1000 dilution. The chemical inhibitors used in this study, U73122 (cat# HY-13419), Gefitinib [28] (cat# HY–50895), and LY294002 [28] (cat# HY–10108), were provided by MedChemExpress (Monmouth Junction, NJ, USA). U73122 is an inhibitor that can specifically inhibit PLC-γ1 phosphorylation at Ser1248 [19,29]. Gefitinib is an EGFR inhibitor that can selectively inhibit EGFR tyrosine kinase activity by binding to the adenosine triphosphate (ATP)-binding site [30]. LY294002 can specifically inhibit Akt phosphorylation by inactivation of its direct activator phosphoinositide 3-kinases (PI3K) [31].

### 2.3. Cell Viability Assay

Cell viability was assessed by using a Trypan blue exclusion test, as described by Fiorito et al. [32,33], with modifications. In brief, either A549 or MDBK cells were seeded into flat 24-well plates and incubated overnight. Confluent cells were exposed to either a DMSO control or the individual chemicals specified for the time length indicated. The cells were collected after trypsinization, and an aliquot of the cell suspension was mixed with an equal volume of 0.4% Trypan-blue (0.4%) (Bio-Rad, Hercules, CA, USA, #1450021). After 10 min, cell numbers were counted by using a Burker chamber under a light microscope. The percentage of cell viability in the chemical treatment groups was calculated by normalization of the number of live cells to that in the control samples. The cell viability of the mock treated control was arbitrarily assigned as 100%.

### 2.4. Virus Replication Inhibition Assay

Confluent A549 or MDBK cells in 24-well plates were pretreated with either DMSO control or indicated inhibitors (Gefitinib, U73122, and LY294002) for 2 h before infection. Cells were then infected with BoHV-1 (MOI of 1) in the presence of the DMSO control or indicated inhibitors at the concentrations specified. After inoculation for 2 h, the cells were washed three times using PBS, and fresh DMEM medium containing either DMSO control or indicated inhibitors was added. After infection for the time length indicated, the cell cultures were collected, and viral titers were determined in MDBK cells. The results were expressed as TCID_50_/mL calculated using the Reed–Muench method.

### 2.5. Western Blotting Analysis

A549 cells in 60 mm dishes were mock-infected or infected with BoHV-1 at an MOI of 1 for 24, 36, or 48 h. Cells were lysed with RIPA buffer (1 × PBS, 1% NP-40, 0.5% sodium deoxycholate, and 0.1% SDS) supplemented with a protease inhibitor cocktail. Cell lysates were clarified by centrifugation at 16,000× *g* for 10 min. The clarified supernatant was subjected to Western blotting analysis using the antibodies specified. GAPDH was probed as a protein loading control. The intensity of the detected protein bands was quantitatively analyzed with the free software ImageJ (https://imagej.nih.gov/ij/download.html), and was normalized to the protein loading control; each analysis was compared with that of the uninfected control, which was arbitrarily set as 1.

## 3. Results

### 3.1. BoHV-1 Productive Infection in Cell Culture Leads to EGFR Activation

**Figure 1:**
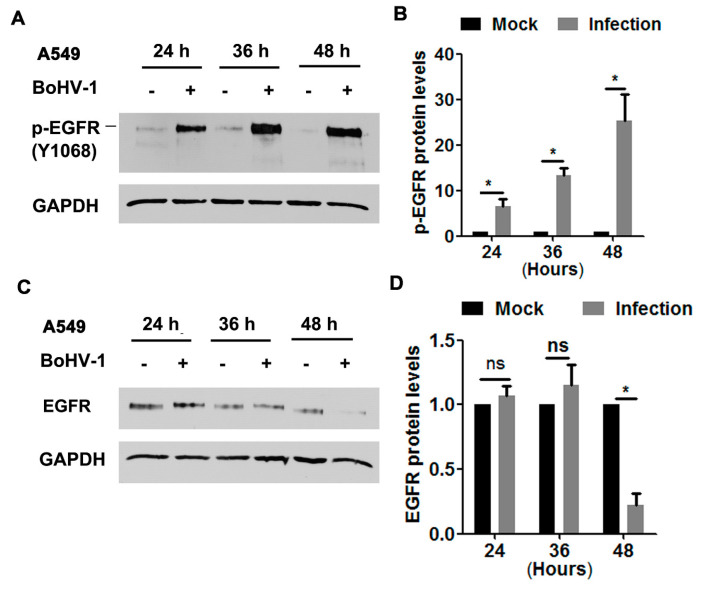
BoHV-1 infection in A549 cells stimulated EGFR phosphorylation (**A**,**C**) Confluent A549 cells in 60 mm dishes were infected with BoHV-1 at an MOI of 1. After infection for 24, 36, or 48 h, cell lysates were analyzed by Western blotting to detect phosphorylated-EGFR(Y1068) (**A**) and EGFR (**C**). Data are representative of three independent experiments. (**B**,**D**) The relative band intensity was analyzed with software ImageJ, and each analysis was compared with that of an uninfected control, which was arbitrarily set as 1. Significance was assessed with a Student’s *t*-test (* *p* < 0.05); ns: not significant.

In order to characterize whether EGFR was activated during infection of A549 cells, protein levels of phospho-EGFR at Tyr1068 (Y1068), a known inducible autophosphorylation site correlated with EGFR kinase activity, was detected via Western blot at 24, 36, and 48 hpi, as determined elsewhere [15]. We found that the levels of phospho-EGFR(Y1068) were dramatically elevated following BoHV-1 infection at all time points sampled (Figure 1A). Quantitative analysis indicated that phospho-EGFR(Y1068) levels increased approximately 6.5, 13.3, and 25.3-fold after infection for 24, 36, and 48 h, respectively (Figure 1B). Steady-state EGFR protein levels were not affected at 24 and 36 h post-infection (hpi), but after infection for 48 h they were decreased to approximately 20% relative to the uninfected control (Figure 1C,D). This depletion of EGFR at 48 hpi may reflect the virus host shutoff function. These results suggest that BoHV-1 infection stimulated EGFR activation, which was not dependent on the steady-state EGFR protein levels.

**Figure 2:**
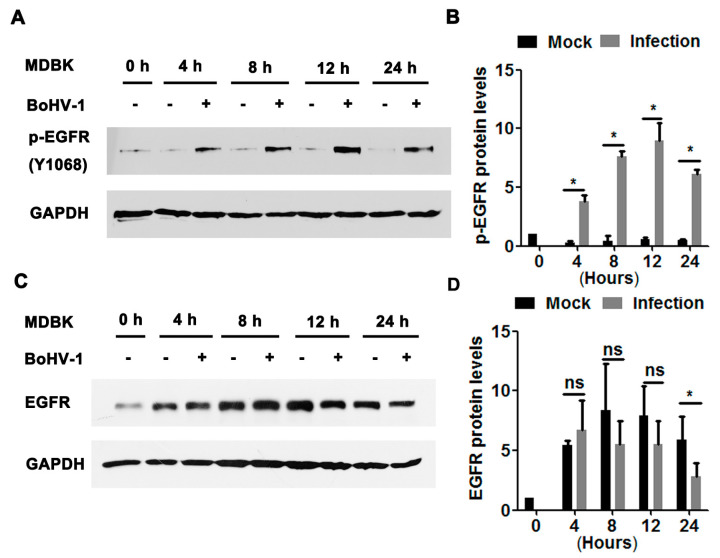
BoHV-1 productive infection in MDBK cells stimulated EGFR phosphorylation. (**A**,**C**) Confluent MDBK cells in 60 mm dishes were infected with BoHV-1 at an MOI of 1. After infection for 4, 8, 12, or 48 h, cell lysates were analyzed by Western blotting to detect phosphorylated-EGFR(Y1068) (**A**) and EGFR (**C**). Data is representative of two or three independent experiments. (**B**,**D**) The relative band intensity was analyzed with Image J software, and each analysis was compared with that of the uninfected control, which was arbitrarily set as 1. Significance was assessed with a Student’s *t*-test (* *p* < 0.05); ns: not significant.

We further explored the effects of BoHV-1 productive infection on EGFR signaling in bovine kidney cells (MDBK cells). As can be seen in Figure 2A, sustained activation of EGFR was stimulated during virus infection in MDBK cells, with phospho-EGFR(Y1068) protein levels increased to approximately 3.8-, 7.6-, 8.9-, and 6.1-fold relative to the uninfected control at 4, 8, 12, and 24 hpi, respectively (Figure 2B). Steady-state EGFR protein levels were significantly decreased at 24 hpi (Figure 2C), reduced to approximately 50% relative to the uninfected control (Figure 2D). In addition, relative to the mock-infected cells at 0 h, steady-state EGFR protein levels in the uninfected cells were consistently increased more than 4-fold from 4 to 24 h. It is probable that higher levels of EGFR were induced to overcome the adverse effects of serum starvation. These data suggest that BoHV-1 infection in MDBK cells also leads to the activation of EGFR, with a similar trend observed in virus-infected A549 cells.

### 3.2. EGFR Inhibitor Decreased BoHV-1 Infection in Cell Culture

**Figure 3:**
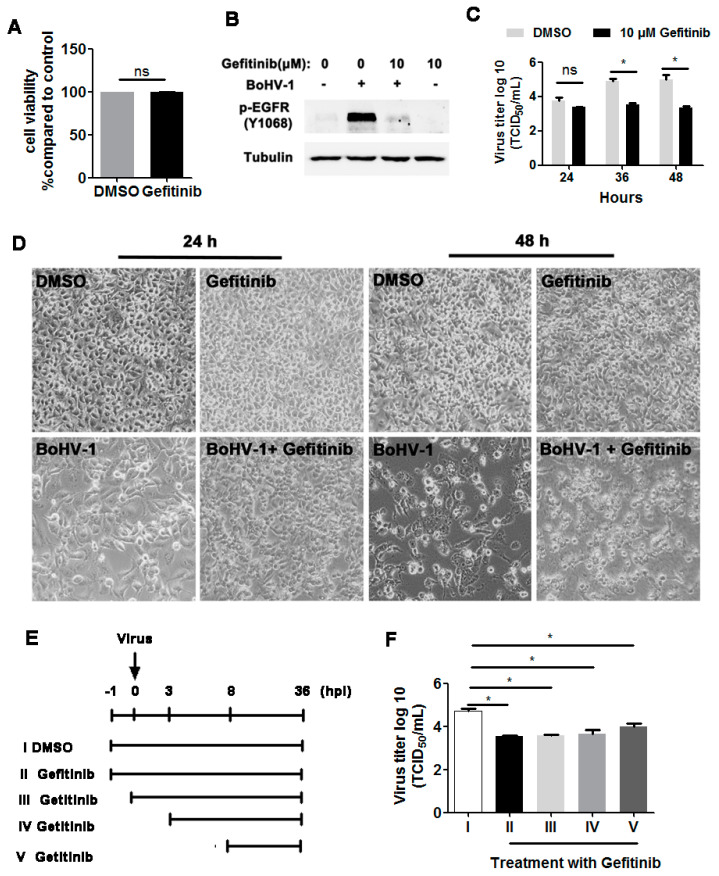
The EGFR inhibitor Gefitinib affected BoHV-1 replication in A549 cells. (**A**) A549 cells in 24-well plates were treated with Gefitinib at a concentration of 10 μM for 48 h. The cytotoxicity of Gefitinib was then analyzed by the Trypan-blue exclusion test. (**B**) A549 cells in 60 mm dishes pretreated with either the DMSO control or Gefitinib (10 μM) were infected with BoHV-1 (MOI = 1) in the presence of DMSO control or Gefitinib, respectively. After infection for 36 h, the cell lysates were prepared and p-EGFR(Y1068) was detected by Western blot. (**C**) A549 cells in 24-well plates pretreated with either DMSO control or Gefitinib (10 μM) were infected with BoHV-1 (MOI = 1) in the presence of DMSO control or Gefitinib, respectively. After infection for 24, 36, or 48 h, the cell cultures were collected and virus titers were determined in MDBK cells. (**D**) A549 cells in 24-well plates were infected with BoHV-1 (MOI = 1) for 24 and 48 h, with or without Gefitinib (10 μM) treatment. The cell morphology was observed under a light microscope. Images shown are representative of two independent experiments (magnification: 200×). (**E**) Diagram showing five different experimental conditions in the time-of-addition assay: (I) DMSO treatment from −1 to 36 hpi, (II) Gefitinib treatment from −1 to 36 hpi, (III) Gefitinib treatment from 0 to 36 hpi, (IV) Gefitinib treatment from 3 to 36 hpi, and (V) Gefitinib treatment from 8 to 36 hpi. (**F**) Viral titer for the time-of-addition assay. Results are the mean of three independent experiments, with error bars showing standard deviations. Significance was assessed with student *t*-test (* *p* < 0.05); ns: not significant.

As our previous results demonstrated that BoHV-1 productive infection led to activation of EGFR in both A549 and MDBK cells, we next investigated whether EGFR signaling played an important role in virus infection. To address this question, we treated virus-infected A549 cells with the chemical inhibitor Gefitinib, a known selective inhibitor of EGFR tyrosine kinase [30]. We first confirmed that Gefitinib at a concentration of 10 μM did not show apparent cytotoxicity in A549 cells after treatment for 48 h ([15] and Figure 3A,D upper panel). As expected, 10 μM Gefitinib significantly inhibited the activation of EGFR induced by virus infection at 36 hpi (Figure 3B) Relative to the DMSO control, 10 μM Gefitinib had no observable effects on virus replication at 24 hpi, but at 36 and 48 hpi the virus titers were reduced by approximately 1.4- and 1.7-logs, respectively (Figure 3C). Ten µM Gefitinib moderately reduced virus infection-induced cytopathology effects at 24 and 48 hpi (Figure 3D, lower panel), which confirmed that the reduced virus yield by Gefitinib was not due to cytotoxicity in A549 cells. A time-of-addition assay indicated that when 10 μM Gefitinib was added at 0, 3, or 8  hpi, approximately 10-fold less virus production was consistently observed (Figure 3E,F). Collectively, these data show that an EGFR inhibitor inhibited BoHV-1 titers in A549 cells, modulating the virus post-entry stage(s).

**Figure 4:**
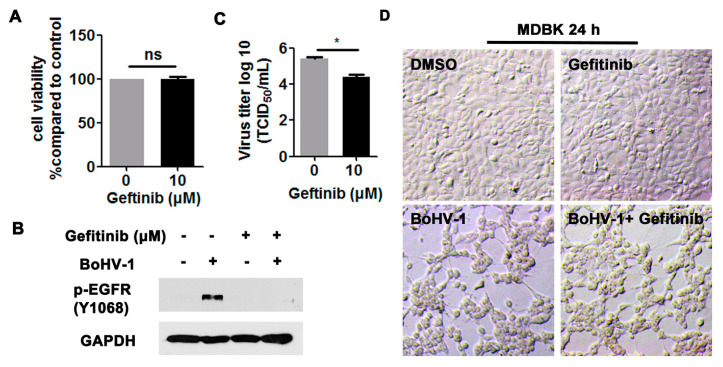
The EGFR inhibitor Gefitinib affected BoHV-1 replication in MDBK cells. (**A**) MDBK cells in 24-well plates were treated with Gefitinib at a concentration of 10 μM for 24 h. The cytotoxicity of Gefitinib was then analyzed by a Trypan-blue exclusion test. (**B**) MDBK cells in 60 mm dishes pretreated with either DMSO control or Gefitinib (10 μM) were infected with BoHV-1 (MOI = 1) in the presence of DMSO control or Gefitinib, respectively. After infection for 24 h, cell lysates were prepared and p-EGFR(Y1068) was detected by Western blot. (**C**) MDBK cells in 24-well plates pretreated with either DMSO control or Gefitinib (10 μM) were infected with BoHV-1 (MOI = 1) in the presence of DMSO control or Gefitinib, respectively. After infection for 24 h, cell cultures were collected and virus titers were determined in MDBK cells. Results are the mean of three independent experiments, with error bars showing standard deviations. Significance was assessed with a Student’s *t*-test (* *p* < 0.05); ns: not significant. (**D**) MDBK cells in 24-well plates, either with or without BoHV-1 infection (MOI = 1), were treated with Gefitinib (10 μM) for 24 h. The cell morphology was observed under a light microscope. Images shown are representative of two independent experiments (magnification: 200×).

We then investigated the effects of EGFR on virus infection in MDBK cells. Ten μM Gefitinib showed no cytotoxicity to MDBK cells after treatment for 24 h (Figure 4A,D, upper panel). As expected, the activation of EGFR induced by virus infection at 24 hpi was significantly blocked by 10 μM Gefitinib (Figure 4B). After infection for 24 h, virus titers were significantly inhibited by 10 μM Gefitinib, which was reduced by approximately 1-log, as detected in comparison to that of the MDSO control (Figure 4C). When the cell morphology was examined, 10 µM Gefitinib had minor effects on alleviating the virus infection-induced cytopathology effects in MDBK cells (Figure 4D, lower panel), supporting that the reduced viral titers in Gefitinib-treated cells were not due to the cytotoxicity to MDBK cells. Overall, these results indicate that BoHV-1 infection leads to the activation of EGFR in both A549 and MDBK cells, which in turn plays an important role in virus infection.

### 3.3. Potential Involvement of EGFR Signaling in the Activation of PLC-γ1 by BoHV-1 Infection in A549 Cells

**Figure 5:**
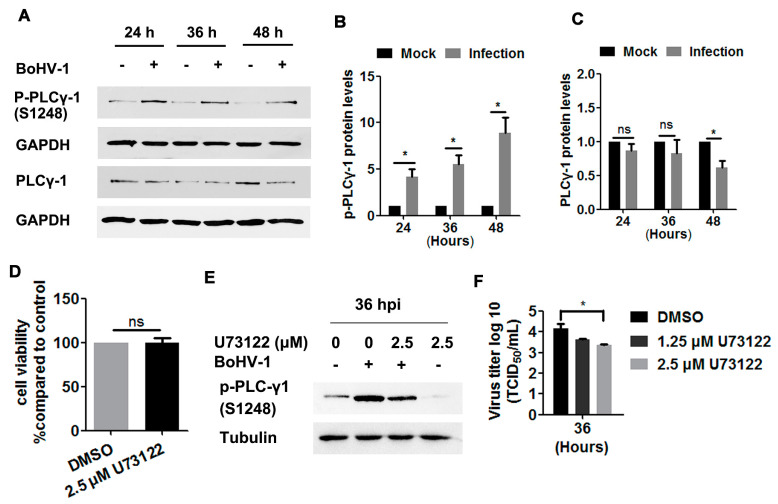
BoHV-1 infection in A549 cells stimulated PLC-γ1 signaling. (**A**) A549 cells in 60 mm dishes were mock-infected or infected with BoHV-1 (MOI = 1) for 24, 36, or 48 h. The cell lysates were then prepared for Western blots to detect p-PLC-γ1(S1248), PLC-γ1, and GAPDH. (**B**,**C**) The relative band intensity of both p-PLC-γ1(S1248) (**B**) and PLC-γ1 (**C**) was analyzed with ImageJ software, and each analysis was compared with that of the uninfected control which was arbitrarily set as 1. (**D**) A549 cells in 24-well plates were treated with U73122 at a concentration of 2.5 μM for 48 h. Cytotoxicity was then analyzed by a Trypan-blue exclusion test. (**E**) A549 cells in 60 mm dishes pretreated with either DMSO control or U73122 (2.5 μM) were infected with BoHV-1 (MOI = 1) in the presence of a DMSO control or U73122, respectively. After infection for 36 h, the cell lysates were prepared and p- PLC-γ1(S1248) was detected by Western blot. (**F**) A549 cells in 24-well plates pretreated with either DMSO control or U73122 (2.5 μM) were infected with BoHV-1 (MOI = 1) in the presence of DMSO control or U73122, respectively. After infection for 36 h, cell cultures were collected and virus titers were determined in MDBK cells. Results are the mean of three independent experiments, with error bars showing standard deviations. Significance was assessed with a Student’s *t*-test (* *p* < 0.05); ns: not significant.

**Figure 6:**
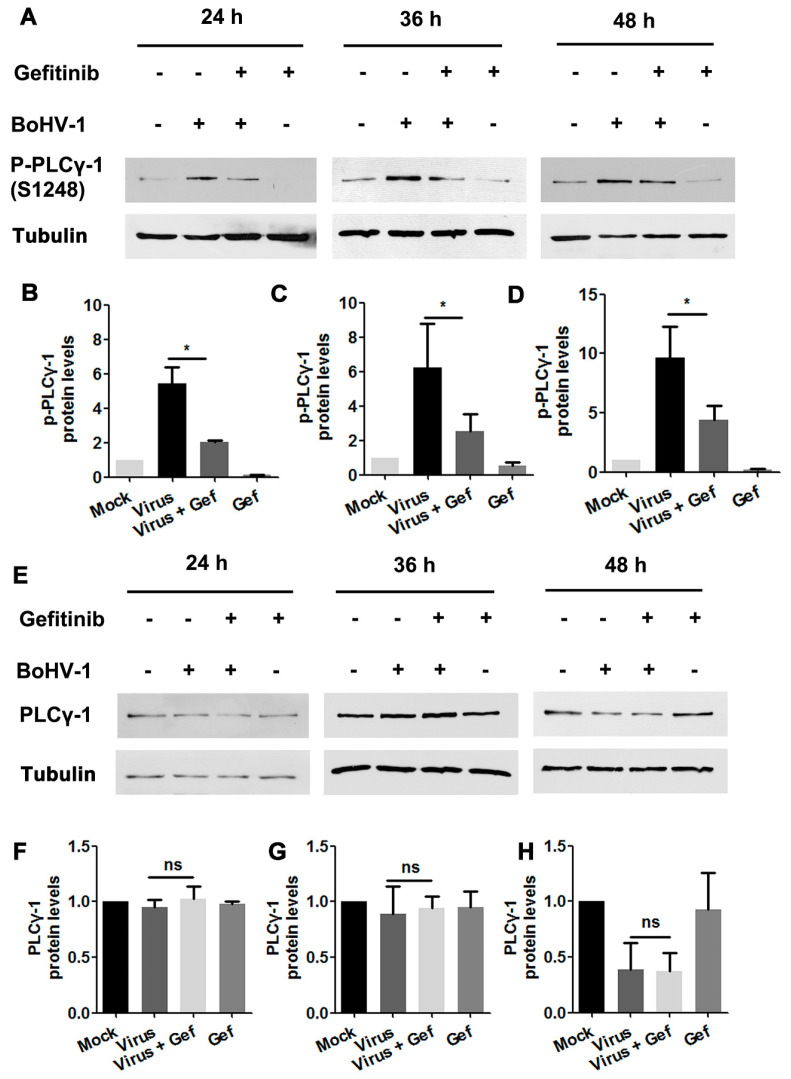
The activation of PLC-γ1 signaling in BoHV-1-infected A549 cells is inhibited by EGFR inhibitor Gefitinib. (**A**) A549 cells in 60 mm dishes pretreated with either DMSO or Gefitinib (10 μM) were infected with BoHV-1 (MOI = 1) in the presence of the indicated chemical. After infection for 24, 36, or 48 h, cell lysates were prepared and subjected to Western blot to detect p-PLC-γ1(S1248) (**A**) and PLC-γ1 (**E**), respectively. (**B**–**D**, **F**–**H**) The relative band intensity was analyzed with ImageJ software, and each analysis was compared with that of the uninfected control, which was arbitrarily set as 1. Data shown are representative of three independent experiments. Significance was assessed with a Student’s *t*-test (* *p* < 0.05).

To more closely investigate the EGFR signaling pathway, we next examined the phosphorylation levels of PLC-γ1 at S1248 in BoHV-1-infected A549 cells, and found these levels to be consistently increased at 24, 36, and 48 hpi (Figure 5A). Quantitative analysis indicated that the levels of PLC-γ1(S1248) were increased by approximately 4-, 5.5-, and 8.9-fold relative to the control, as detected at 24, 36, and 48 hpi, respectively (Figure 5B). PLC-γ1 steady-state protein levels were not affected until after infection for 48 h (Figure 6A), which at this time point were reduced to approximately 60% relative to the control (Figure 6C). Taken together, the increased phosphorylation levels of PLC-γ1 attributed to virus infection were not due to increased PLC-γ1 steady-state protein levels, supporting that BoHV-1 productive infection in A549 cells led to sustained activation of PLC-γ1 at 24, 36, and 48 hpi.

We then investigated whether PLC-γ1 signaling affected BoHV-1 replication in A549 cells, using the PLC-γ1 specific inhibitor U73122 as reported previously [19,29,34]. U73122 at a concentration of 2.5 μM did not show overt cytotoxicity in A549 cells after incubation for 48 h (Figure 5D). The phosphorylation of PLC-γ1 at S1248 induced by virus infection was significantly blocked at 36 hpi (Figure 5E), suggesting that U73122 could inhibit the activity of PLC-γ1. Relative to the DMSO control, 2.5 μM U73122 inhibited virus titers at 36 hpi, with titers reduced by ~1 log (Figure 5F). These results suggest that BoHV-1 productive infection in A549 cells leads to the activation of PLC-γ1, which is important for efficient viral infection.

To investigate whether BoHV-1 infection stimulated PLC-γ1 through EGFR, virus-infected A549 cells were treated with either DMSO control or the EGFR inhibitor Gefitinib. Total cell lysates were then analyzed by Western blotting to detect the variation of PLC-γ1(S1248). We found that virus infection-stimulated PLC-γ1 was significantly inhibited by Gefitinib (Figure 6A). Quantitative analysis indicated that PLC-γ1(S1248) levels were consistently reduced to more than 50% relative to the control, detected at 24, 36, and 48 hpi, respectively (Figure 6B–D). In the context of virus infection, Gefitinib showed no effect on PLC-γ1 steady-state protein expression at any time point examined (Figure 6E–H), suggesting that the decreased PLC-γ1(S1248) levels by Gefitinib were not due to the reduction of total PLC-γ1 expression. These data indicate a potential role of EGFR in the activation of PLC-γ1 attributed to BoHV-1 infection.

### 3.4. The Activation of Akt Signaling in BoHV-1-Infected A549 Cells is Independent of EGFR

**Figure 7:**
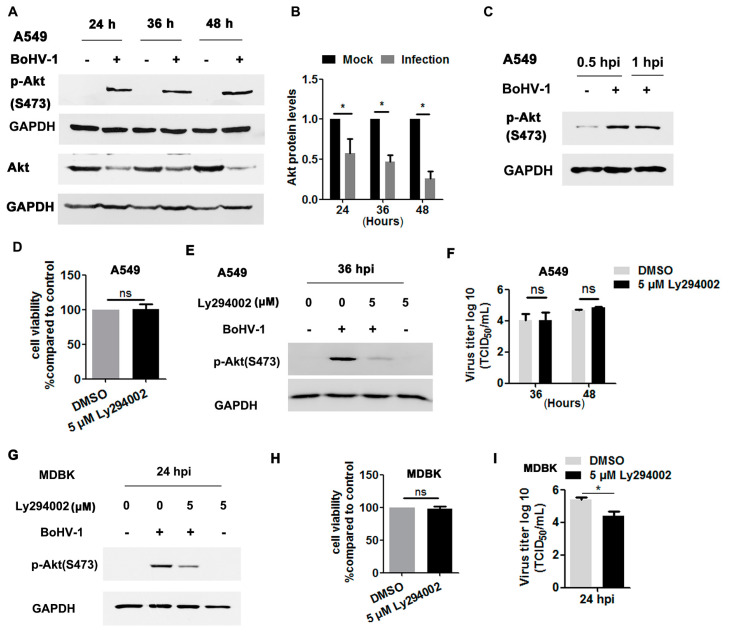
BoHV-1 infection stimulated Akt for efficient replication in a cell type-specific manner. (**A**) A549 cells in 60 mm dishes were mock-infected or infected with BoHV-1 (MOI = 1) for 24, 36, or 48 h. The cell lysates were prepared for Western blot to detect p-Akt(S473) and Akt. (**B**) The relative band intensity of Akt was analyzed with ImageJ software, and each analysis was compared with that of uninfected control, which was arbitrarily set as 1. (**C**) A549 cells in 60 mm dishes were mock-infected or infected with BoHV-1 (MOI = 1) for 0.5 and 1 h. The cell lysates were then prepared for Western blot to detect p-Akt(S473). (**D**,**H**) Either A549 or MDBK cells in 24-well plates were treated with Ly294002 at a concentration of 5 μM for 48 and 24 h, respectively. The cytotoxicity was then analyzed by a Trypan-blue exclusion test. (**E**) A549 cells in 60 mm dishes pretreated with either DMSO control or Ly294002 (5 μM) were infected with BoHV-1 (MOI = 1) in the presence of a DMSO control or Ly294002, respectively. After infection for 36 h, cell lysates were prepared and p-Akt(S473) was detected by Western blot. (**F**) A549 cells in 24-well plates pretreated with either a DMSO control or Ly294002 (5 μM) were infected with BoHV-1 (MOI = 1) in the presence of DMSO or Ly294002, respectively. At 36 and 48 hpi, cell cultures were collected and virus titers were determined in MDBK cells. (**G**) MDBK cells in 60 mm dishes pretreated with either the DMSO control or Ly294002 (5 μM) were infected with BoHV-1 (MOI = 1) in the presence of DMSO control or Ly294002, respectively. After infection for 24 h, the cell lysates were prepared and p-Akt(S473) was detected by Western blot. (**I**) MDBK cells in 24-well plates pretreated with either the DMSO control or Ly294002 (5 μM) were infected with BoHV-1 (MOI = 1) in the presence of DMSO or Ly294002, respectively. At 24 hpi, cell cultures were collected and virus titers were determined in MDBK cells. Data and error bars denote the variability between three independent experiments. Significance was assessed with a Student’s *t*-test (* *p* < 0.05); ns: not significant.

Akt, a canonical downstream effector of the EGFR pathway, is important for BoHV-1 infection in MDBK cells [35,36,37], but its role during virus infection in A549 cells has been unknown. To address this question, we initially detected whether Akt signaling was activated in BoHV-1-infected A549 cells. As can be seen in Figure 7A, the phosphorylation of Akt at S473 was consistently enhanced at 24, 36 and 48 hpi. However, the levels of total Akt gradually decreased following virus infection, with reductions to 57.6%, 46.8%, and 25.6% at 24, 36, and 48 hpi, respectively, compared to uninfected cells (Figure 7A,B). These results support that virus infection in A549 cells also stimulates Akt signaling. As observed in virus-infected MDBK cells [37], Akt was activated after infection for 0.5 and 1.0 h (Figure 7C).

We then investigated whether Akt signaling was necessary for BoHV-1 replication in A549 cells using the Akt-specific inhibitor Ly294002. Five μM Ly294002 did not show cytotoxicity in A549 cells after treatment for 48 h (Figure 7D). As expected, the activation of Akt induced by virus infection at 36 hpi was significantly blocked by 5 μM Ly294002 (Figure 7E). Relative to the DMSO control, 5 μM Ly294002 did not show inhibitory effects on virus titers detected at either 36 or 48 hpi (Figure 7F). Consistent with our previous report that BoHV-1 infection in MDBK cells stimulated Akt at 24 hpi [37] (Figure 7G). Ly294002 at a concentration of 5 μM showed no cytotoxicity to MDBK cells (Figure 7F), but it significantly inhibited the activation of Akt stimulated by virus infection and BoHV-1 titers, as detected at 24 hpi (Figure 7G,I). Taken together, these data show that BoHV-1 infection led to activation of Akt during infection of both A549 cells and MDBK cells, but was important for virus infection in MDBK cells but not in A549 cells, suggesting that Akt signaling affected BoHV-1 infection in cell type-specific manner.

**Figure 8:**
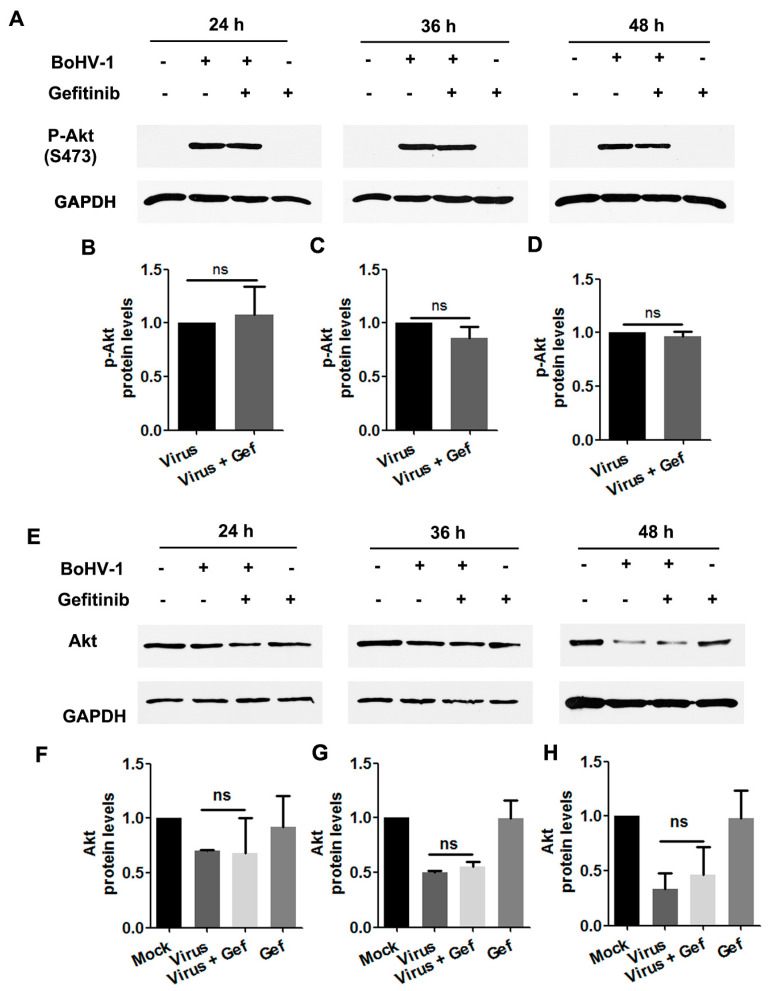
Activated Akt stimulated by BoHV-1 infection in A549 cells was inhibited by the EGFR inhibitor Gefitinib. (**A**) A549 cells in 60 mm dishes pretreated with either DMSO or Gefitinib (10 μM) were infected with BoHV-1 (MOI = 1) in the presence of the chemical indicated. After infection for 24, 36, or 48 h, cell lysates were prepared and subjected to Western blot to detect p-Akt(S473) (**A**) and Akt (**E**). (**B**–**D**) and (**F**–**H**) The relative band intensity was analyzed with ImageJ software, and each analysis was compared with that of the uninfected control which was arbitrarily set as 1. Data shown are representative of two or three independent experiments. Significance was assessed with a Student’s *t*-test; ns, not significant.

To characterize whether Akt was stimulated during BoHV-1 infection through EGFR, virus-infected A549 cells were treated with the EGFR specific inhibitor Gefitinib throughout infection, and levels of Akt(S473) were assessed by Western blot. We found that in virus-infected cells, treatment with Gefitinib did not show inhibitory effects on either the levels of Akt(S473) or the steady-state Akt protein (Figure 8A–H). Collectively, these data provide evidence that in BoHV-1-infected A549 cells, activation of Akt was not completely dependent on the canonical upstream activator EGFR. An EGFR-independent mechanism for the activation of Akt might be employed in virus-infected A549 cells.

## 4. Discussion

Accumulated studies have demonstrated that a large number of viruses usurp EGFR signaling with diverse mechanisms to build a favorable cellular environment for efficient entry, as reviewed previously [38]. For example, EGFR acts as a co-receptor for the entry of both human cytomegalovirus (HCMV) and adeno-associated virus 6 (AAV6) [39,40], and the EGFR/PI3K/MEK/Rock/cofilin cascade is manipulated by HSV-1 to promote F-actin polymerization for efficient entry into neuronal cells [41]. Prior studies in the literature are generally in agreement that EGFR plays a critical role in the entry stages for most viruses [16,38]. As such, we hypothesized that EGFR-mediated virus entry represents a versatile mechanism that might also apply to BoHV-1. Here, we reported that BoHV-1 infection activates EGFR signaling in two cell types (MDBK and A549 cells), which in turn play an important role in the virus infection (Figure 1, Figure 2, Figure 3 and Figure 4). To further our understanding of the role of EGFR signaling during virus infection, we performed a time-of-addition assay, and found that post-entry stages were also affected by the EGFR-specific inhibitor Gefitinib during infection of A549 cells (Figure 3). This result was not surprising, as it has been reported previously that upon stimulation, activated EGFR can translocate into the nucleus, where it acts as a transcription factor by binding to and activating AT-rich consensus DNA sequences [42,43], regulating the expression of host genes, such as the peroxisome proliferator-activated receptor γ coactivator-1α (PGC-1α) gene [44,45]. Of note, the BoHV-1 genome possesses an abundance of A–T rich sequences. Whether EGFR could bind directly to the viral genome to regulate pertinent gene transcription is an interesting issue that warrants further study with CHIP-seq. Moreover, viral genome replication and viral particle assembly, as well as virus budding and egress, are potentially affected by EGFR inhibition, an additional area deserving of subsequent investigation.

Ligand binding induces the phosphorylation of EGFR in the conserved cytoplasmic domain having protein–tyrosine kinase activity, which allows multiple adaptor and effector proteins to bind through their Src homology 2 domains, leading to the activation of downstream signaling cascades, including phosphatidylinositide 3-kinases (PI3Ks)/Akt and PLC-γ1, as reviewed previously [38]. In line with this recognized paradigm, we found that virus infection activated PLC-γ1 partially through EGFR, as treatment with the EGFR specific inhibitor Gefitinib led to a significant inhibition of PLC-γ1 phosphorylation in virus-infected cells (Figure 6). In addition, in the presence of Gefitinib, residual PLC-γ1(S1248) levels were approximately two-fold higher than those during mock infection, and Gefitinib could not completely extinguish virus infection (Figure 5). We assumed that either viral proteins or viral nucleic acid may have the capacity to stimulate PLC-γ1 independent of EGFR, which need further studies in the future.

In contrast to the canonical paradigm, Gefitinib did not show inhibitory effects on the activation of Akt attributed to virus infection (Figure 8), an indicator of PI3K activity, suggesting that EGFR signaling is not the exclusive upstream activator of the PI3K/Akt cascade in the context of virus infection. This is not surprising, because the activation of Akt by HSV-1 UL46 encoded proteins has been reported previously [46]. As HSV-1 is genetically close to BoHV-1, it is plausible that selected proteins encoded by BoHV-1 may also have the capacity to activate Akt, which was not inhibited by Gefitinib.

The PI3K/Akt pathway is exploited by multiple viruses for efficient replication (reviewed in [47]). In this study, we show that Ly294002, a widely used Akt-specific inhibitor, inhibited BoHV-1 titers in MDBK cells but not in A549 cells (Figure 7F,I), suggesting that Akt affects BoHV-1 infection in a cell type-specific manner. The established bovine cell line MDBK is derived from the kidney of an apparently normal adult Bos Taurus, while the human cell line A549 was generated through explant culture of lung carcinomatous tissue [48]. The diverse origins (human vs. Bos Taurus and lung vs. kidney) and biological characters (carcinoma vs. normal) of these two cell cultures may partially account for the distinct effects of Akt activation during virus replication. Interestingly, this is not the first report to show that PI3K/Akt signaling had no effect on virus efficient infection, as it has been previously reported that Ly294002 had no effects on porcine reproductive and respiratory syndrome virus (PRRSV) propagation in MARC-145 cells [49]. Here, for the first time, we show that Akt-specific inhibitor LY294002 has effects on BoHV-1 infection in a cell type-dependent manner. Considering that pro-survival cellular signaling pathways are generally important for either the establishment or maintenance of BoHV-1 latency, and that EGFR is also expressed in the trigeminal ganglion [50], a critical site for establishment of BoHV-1 latency and reactivation cycles, subsequent study of the role of EGFR in BoHV-1 latency- reactivation cycles in vivo is warranted.

BoHV-1 represents a promising oncolytic vector, having the capacity to infect a diversity of human tumor cells, including A549 cells [7,9,51,52]. Currently, the mechanism(s) underlying the oncolytic effects and how tumor cells support virus infection is poorly understood. In this study, we show for the first time that the EGFR/PLC-γ1 cascade is activated during BoHV-1 virus infection in A549 cells for efficient replication (Figure 1, Figure 3, Figure 5, and Figure 6). Although Akt signaling is activated in virus-infected A549 cells, it is not necessary for efficient virus replication (Figure 7F). These data add to our knowledge of how EGFR signaling transduction affects productive virus infection in A549 cells, which contributes to our understanding of the oncolytic effects of this virus. So far, great effort has been directed toward developing anticancer agents with the capacity to interfere with EGFR activity, based on the theory that EGFR signaling is important for the progression of diverse cancers [53,54]. Here, we show that even though the pro-survival signaling EGFR was activated due to BoHV-1 infection in A549 cells, cell death was still induced (Figure 3D). These results support that BoHV-1 oncolytic effects do not follow the recognized paradigm that an agent having anti-EGFR activity is ideal for anticancer treatment. Of note, during virus infection, numerous viral products, including virus protein and nucleic acid, were abundantly introduced into the cells. These viral products may cooperatively account for this discrepancy. Therefore, there is a need to more closely study the mechanism(s) governing the oncolytic effects of BoHV-1 and their cell type-specific inhibition of EGFR activity.

## 5. Conclusions

In summary, in this study, for the first time we provide evidence that BoHV-1 infection stimulates the EGFR/PLC-γ1 cascade in A549 cells, which in turn is important for efficient virus replication. Though Akt is stimulated in both BoHV-1-infected A549 cells and MDBK cells, it modulates BoHV-1 infection in a cell type-specific manner. These novel findings contribute to our understanding of how EGFR and its canonical downstream effectors (PLC-γ1 and Akt) are manipulated during BoHV-1 productive infection in diverse cell types. Furthermore, we found that even though pro-survival EGFR signaling was activated in A549 cells following BoHV-1 infection, cell death was still induced, supporting that increased EGFR activity is not positively associated with BoHV-1 oncolytic effects.
